# Looking Beyond the Obvious: An Uncommon Intermammary Groove-Adjacent Breast Cancer Found on Screening Mammography

**DOI:** 10.7759/cureus.77367

**Published:** 2025-01-13

**Authors:** Rita C Constantino, Sara Almeida, Bárbara Junqueira, Patrícia C Carreira, Mariana Coutinho

**Affiliations:** 1 Planalto Family Health Unit, Leziria Local Health Unit, Santarém, PRT; 2 Terra Viva Family Health Unit, Leziria Local Health Unit, Cartaxo, PRT

**Keywords:** breast cancer, early detection, intermammary, rare location, screening

## Abstract

Breast cancer exhibits a high prevalence among women and represents a significant contributor to cancer-related mortality. Routine screening substantially reduces mortality rates by enabling the early detection of tumors. This report describes an uncommon case of breast cancer located deep near the intermammary groove in a 63-year-old woman. The tumor’s asymptomatic presentation, small size, and atypical location posed a diagnostic challenge. However, screening mammography revealed a suspicious axillary adenopathy, prompting further evaluation. MRI identified a 6 mm intermammary groove-adjacent lesion, and a biopsy confirmed the cancer diagnosis. Staging excluded metastasis. The patient underwent neoadjuvant chemotherapy, and nearly a year later, MRI imaging showed total remission. Sentinel lymph node biopsy was negative for breast cancer, suggesting a favorable prognosis. This case highlights the importance of primary care, promoting screening programs, and early detection in improving prognosis. Enhanced access to screening and meticulous radiological interpretation are critical to optimizing patient outcomes.

## Introduction

Breast cancer is the most frequently diagnosed cancer globally and a leading cause of cancer-related death in women [1,2]. The lifetime risk is one in eight women. In Portugal, in 2022, nearly 9,000 cases were diagnosed, and 2,000 deaths occurred [3]. The main risk factors for developing breast cancer are female sex and age. Additional risks include personal or family history of related cancers and genetic mutations, high breast density, prior high-risk breast biopsies, chest radiotherapy, early menarche, late menopause, few pregnancies, hormone therapy, alcohol consumption, tobacco use, sedentarism, and obesity [4-6].

Breast cancer mortality is decreasing owing to screening programs and advances in therapeutic approaches [2]. Early-stage cancer is more frequent in asymptomatic women (58.1%) [7]. Screening promotes early diagnosis of smaller, less invasive, or asymptomatic tumors, improving treatment outcomes [2,3,7,8].

All major expert groups recommend mammography every two years for asymptomatic average-risk women as it reduces mortality compared to no screening [1,4,8]. There is no evidence supporting screening with clinical or self-examination, ultrasound, and MRI [4,8]. Age recommendations differ, with European guidelines suggesting 50 to 69 years [1], while US guidelines recommend 50 to 74 years [9].

In Portugal, guidelines recommend ages 50 to 69 years [10] and 45 to 74 years in insular regions [11,12]. Digital mammography is performed biennially, mainly in mobile units visiting all municipalities [3]. Primary care physicians counsel and confirm screening adherence and verify the correct management of abnormal findings. Higher primary care interaction improves adherence to regular screening mammography [13].

Screening mammography captures the mobile inferior/lateral and fixed upper/medial breast areas in craniocaudal (CC) and mediolateral oblique (MLO) views. Often, two radiologists review mammograms to improve cancer detection [8]. However, mammography has some disadvantages, including false positives, false negatives, and overdiagnosis [4]. Non-detection occurs in 10-20% of cases but can be reduced by adding ultrasound [4,14]. When both are negative, the malignancy risk is 0-3% [8]. However, a recent literature review found that sensitivity is lower in asymptomatic women (76% vs. 81%), with average sensitivity and specificity being 60% and 80%, respectively [7].

Tumors frequently occur in the upper outer quadrant, in up to 50% of cases, and the upper inner quadrant, in 15% of cases. Lower outer quadrant, central, and lower inner quadrant are less common locations [15,16]. Intermammary breast cancer is rare, with only two reported cases [5,16].

This case report aims to emphasize the importance of screening and early diagnosis, typically promoted by primary care doctors, in improving patient prognosis. It also seeks to discuss the limitations of current screening methods in detecting cancer in uncommon locations and underline the broad spectrum of alterations that can be found in examinations performed for other reasons.

## Case presentation

We present the case of a 63-year-old woman with a personal history of hypertension, dyslipidemia, bronchiectasis, sleep apnea, benign thyroid nodules, lumbar disc hernia, and an L5 lytic lesion (under surveillance and stable). Reproductive history included menarche at age 12, three pregnancies and two live births (at ages 25 and 35 years), breastfeeding for eight months in total, and menopause at 52 years of age, with no hormonal therapy. There was no history of smoking, alcohol abuse, or breast cancer in her family.

Mammography revealed a suspicious adenopathy in the right axilla on the MLO view. Physical examination revealed a palpable 2 cm adenopathy in the right axilla, while the breast examination was normal. Ultrasound of the right axilla detected a 13 × 28 mm suspicious adenopathy, which was subsequently biopsied. The mammary ultrasound was normal. The anatomopathological analysis confirmed ganglionar metastatic disease, compatible with breast origin with positive estrogen receptor (ER) and progesterone receptor (PR) and negative human epidermal growth factor receptor 2 (HER2).

**Figure 1:**
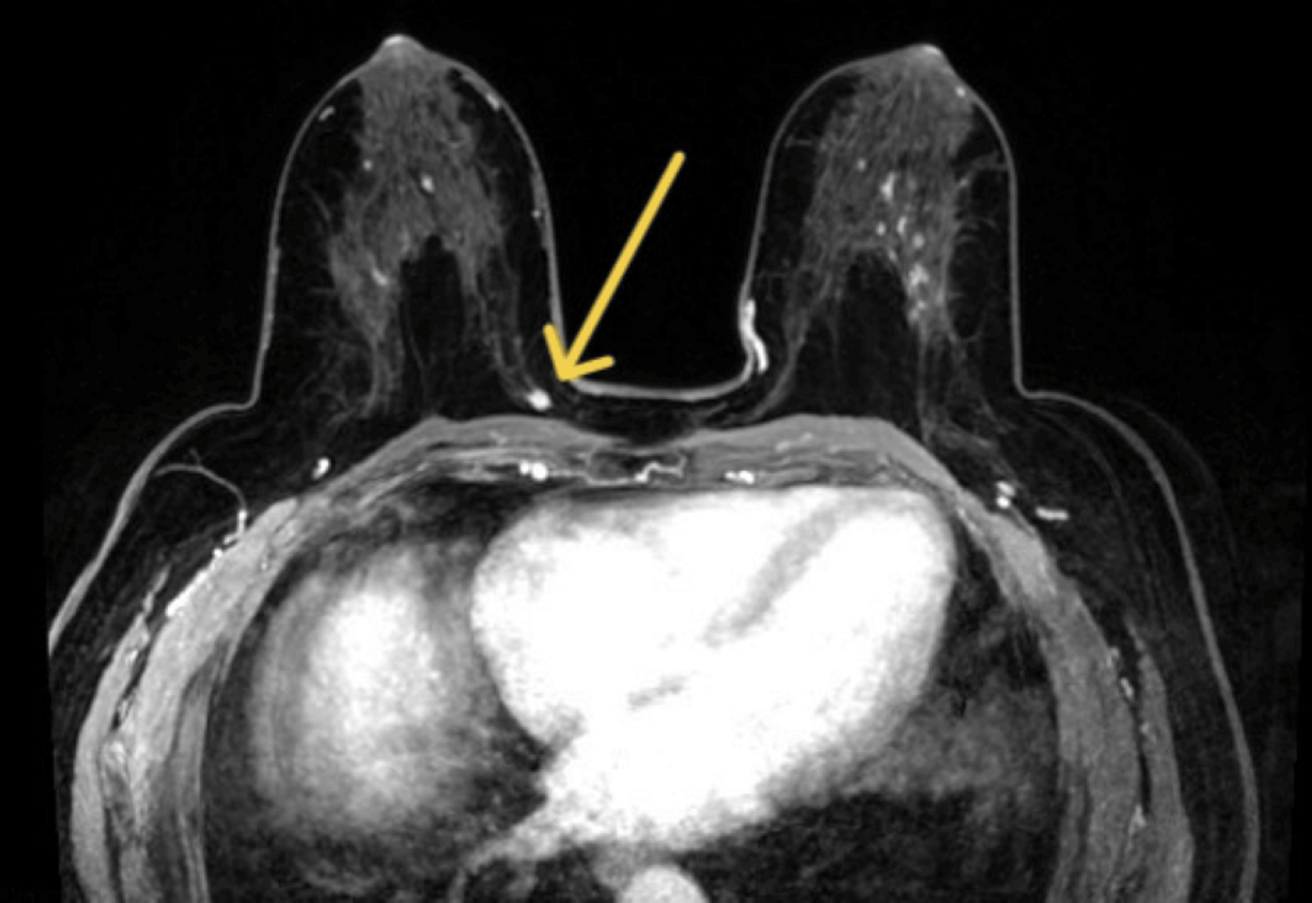
MRI showing the primary tumor (yellow arrow), a 6 mm nucleus between the inner quadrants, in a deep location separated from the thoracic wall by 2 mm, and adjacent to the intermammary groove.

**Figure 2:**
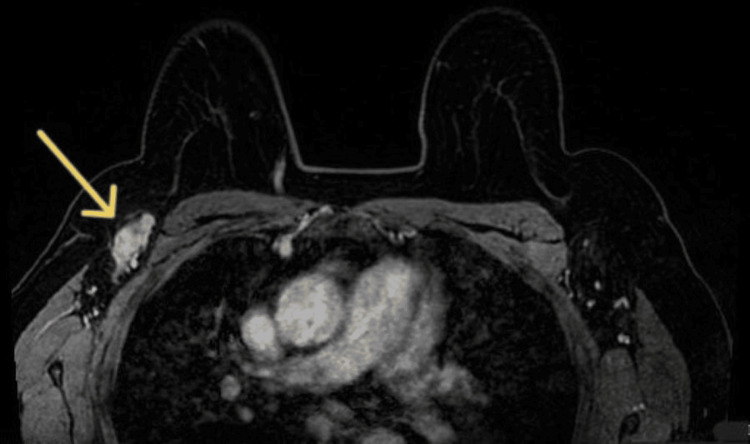
MRI showing suspicious 30 mm adenopathy (yellow arrow) in the right axilla.

The patient attended routine primary care appointments every six months for hypertension management. Shortly after receiving the abnormal result, the family physician contacted the patient and ordered an MRI. The MRI identified the primary tumor, a 6 mm nucleus between the inner quadrants, in a deep location separated from the thoracic wall by 2 mm, and adjacent to the intermammary groove (Figure 1), confirming the presence of a 30 mm suspicious adenopathy in the right axilla (Figure 2) and categorizing the right breast as Breast Imaging-Reporting and Data System (BI-RADS) 4C (highly suspicious).

A biopsy of the 6 mm nucleus revealed an invasive carcinoma of no specific subtype with positive ER and PR and negative HER2. Staging scintigraphy and thoracic, abdominal, and pelvic CT did not identify any other metastases.

**Figure 3:**
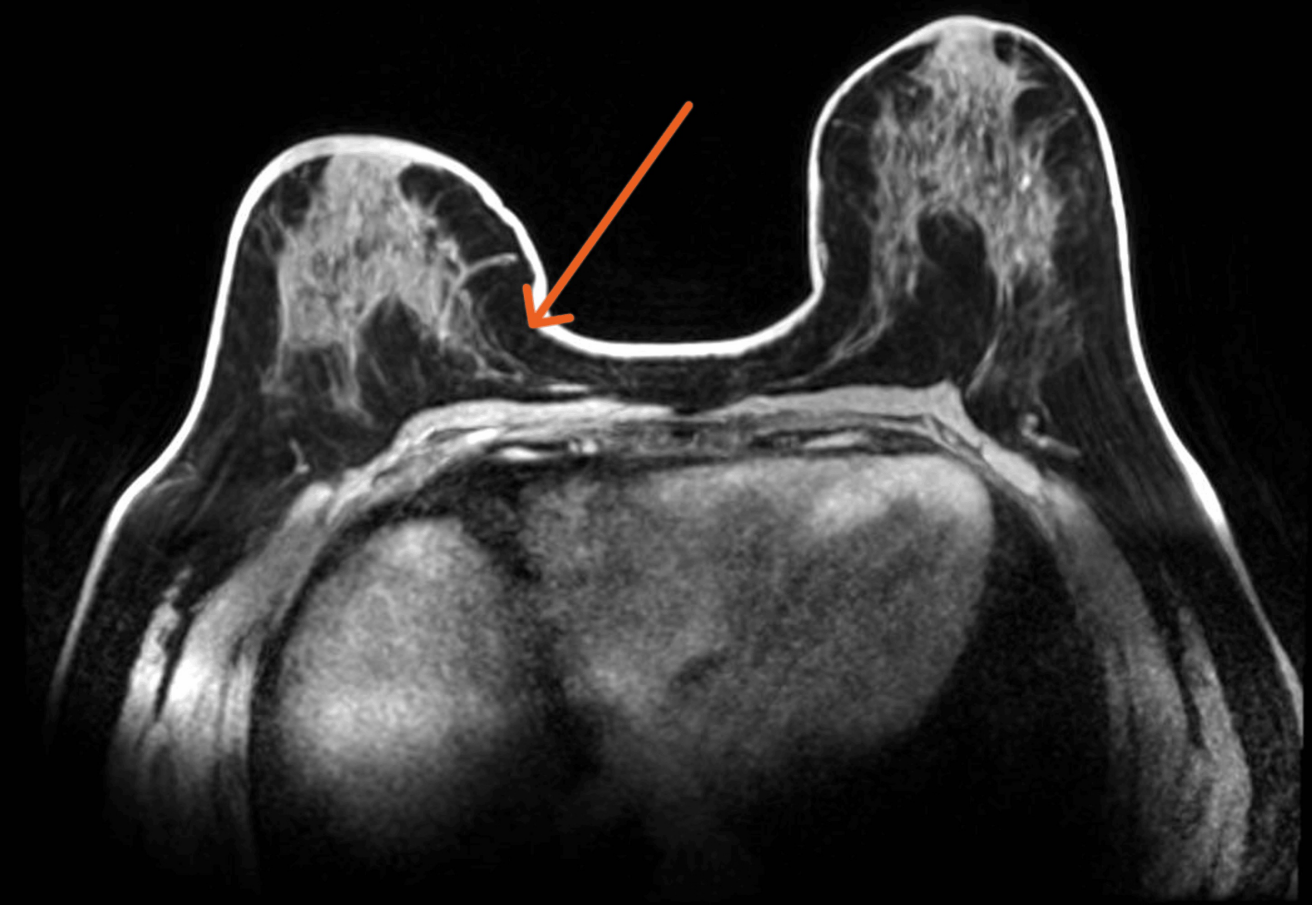
Post-chemotheraphy right breast MRI showing no areas of suspicious enhancement in the area where the primary tumor was previously noted (orange arrow).

**Figure 4:**
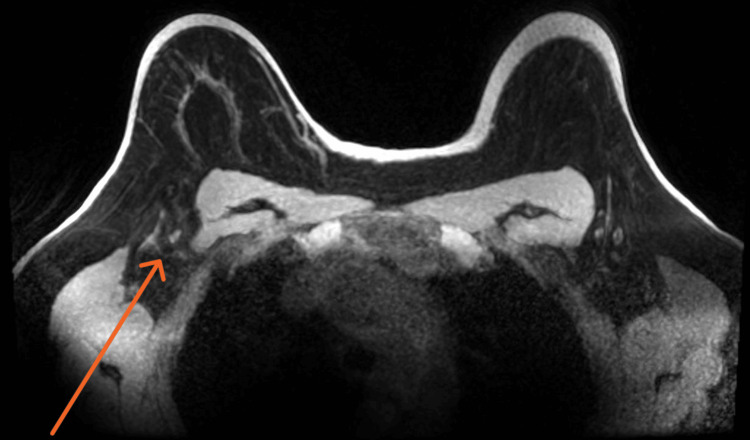
Post-chemotheraphy MRI of right axillary adenopathy (orange arrow) does not show cortical thickening.

Chemotherapy with neoadjuvant intent was initiated with four cycles of doxorubicin and cyclophosphamide every 21 days, followed by 12 cycles of paclitaxel once a week. Following chemotherapy, an MRI revealed no areas of suspicious enhancement in the right breast (Figure 3). Axillary adenopathy showed no signs of cortical thickening (Figure 4), suggesting total remission.

The patient was scheduled for surgery to resect the affected areas and undergo a sentinel lymph node biopsy. However, mammography, ultrasound, and CT imaging performed the same morning as the surgery were unable to find the clip placed on the primary tumor. As a result, surgical resection was performed only in the axillary area. Two sentinel lymph nodes were biopsied, which were negative for breast cancer. The patient will now begin treatment with radio and hormone therapy.

## Discussion

This case is unique due to its unusual location, small size, and asymptomatic presentation. The primary tumor was a 6 mm nucleus present at a deep location near the chest wall between the inner quadrants and adjacent to the intermammary groove. These locations have low tumor incidence [5,15,16].

Considering that large tumors are more easily detected on mammography [7] and that CC and MLO views aim to capture the mobile inferior/lateral and fixed upper/medial breast areas [8], not evaluating deep, peripheral, and intermammary tissue, concerns about the sufficiency of mammography as a screening method can be raised.

In fact, unusual cases highlight the limitations of mammography, in accordance with recent findings that diagnostic accuracy may be lower than previously reported [5,7]. A review and update of the current screening guidelines may be appropriate, pending further investigation and robust evidence on the benefits of alternative screening approaches. For instance, combining mammography with ultrasound increases sensitivity, while MRI is capable of detecting tumors missed by both techniques [7].

In this case, nearly one year after the screening, the prognosis remained favorable. The tumor responded well to chemotherapy, with imaging evidence of regression and sentinel lymph node biopsy negative for breast cancer.

This case highlights the importance of screening as early diagnosis and treatment significantly contribute to a favorable prognosis. By verifying the screening results, the family physician was able to expedite obtaining an MRI that found the primary tumor, emphasizing the crucial role of primary care. Enhancing accessibility to screening is crucial, and mobile screening units may improve program adherence in smaller townships and rural areas [2,3,7,8].

A key factor in this diagnosis was the detection of axillary adenopathy in the MLO mammogram view, an unexpected finding as the axilla is not always visible [8]. Mammograms aim to detect breast abnormalities, but other suspicious signs can be present. This emphasizes that examinations can transcend their intended purpose and that clinicians should maintain a broad approach to exam interpretation.

## Conclusions

This case highlights the importance of screening mammography and the crucial role of primary care. It also shows the importance of primary care physicians and radiologists performing a meticulous radiologic assessment and the relevance of looking beyond the obvious to identify rare and unexpected findings. The tumor’s characteristics and recent evidence that mammography’s accuracy in detecting tumors may be lower than previously reported may lead to a future revision of current breast cancer screening, prompting further investigation into alternative technological screening methods. Considering the favorable impact of screening on prognosis, future efforts should focus on advocating for guideline revisions and enhancing quality and equity in accessing screening programs.
